# Remotely Programmable Deep Brain Stimulator Combined with an Invasive Blood Pressure Monitoring System for a Non-Tethered Rat Model in Hypertension Research

**DOI:** 10.3390/brainsci13030504

**Published:** 2023-03-16

**Authors:** Žilvinas Chomanskis, Vytautas Jonkus, Tadas Danielius, Tomas Paulauskas, Monika Orvydaitė, Kazimieras Melaika, Osvaldas Rukšėnas, Vaiva Hendrixson, Saulius Ročka

**Affiliations:** 1 Clinic of Neurology and Neurosurgery, Institute of Clinical Medicine, Faculty of Medicine, Vilnius University, LT-01513 Vilnius, Lithuania; 2 Department of Neurosurgery, Vilnius University Hospital Santaros Klinikos, LT-08406 Vilnius, Lithuania; 3 Faculty of Physics, Vilnius University, LT-01513 Vilnius, Lithuania; 4 Institute of Applied Mathematics, Faculty of Mathematics and Informatics, Vilnius University, LT-01513 Vilnius, Lithuania; 5 Department of Neurobiology and Biophysics, Institute of Biosciences, Life Sciences Center, Vilnius University, LT-01513 Vilnius, Lithuania; 6 Faculty of Medicine, Vilnius University, LT-01513 Vilnius, Lithuania; 7 Department of Physiology, Biochemistry, Microbiology and Laboratory Medicine, Institute of Biomedical Sciences, Faculty of Medicine, Vilnius University, LT-01513 Vilnius, Lithuania

**Keywords:** deep brain stimulation, caudal ventrolateral medulla, closed-loop stimulation, rat model, hypertension, telemetry

## Abstract

The control circuits of blood pressure have a strong neural regulatory element important in the pathogenesis of essential drug-resistant hypertension. Targeting lower medullary neural control mechanisms of blood pressure by electrical stimulation could be beneficial, and therefore, a novel device is needed. This paper presents a remotely programmable deep brain stimulator with an invasive continuous blood pressure monitoring system in a non-tethered rat model. The device is designed for lower medullary deep brain stimulation research with minimal interference to a daily animal routine. Electrodes were implanted in the caudal ventrolateral medulla. Animal survivability, catheter patency rates, and device data drift were evaluated. Eight out of ten rats survived the surgery and testing period with no or mild temporary neurological compromise. The study revealed that carotid catheters filled with heparinized glycerol ensure better catheter patency rates and blood pressure transduction. There was no significant drift in the device’s pressure sensitivity during the experiment. To our knowledge, this is the first experimental study to show considerable animal survival after lower medullary implantation. Combining the ability to measure and monitor invasive blood pressure with a closed-loop brain pulse generator in a single device could be of potential value in future hemodynamic animal research.

## 1. Introduction

Neuromodulation, in a broad sense, is various invasive and noninvasive techniques that utilize chemical agents, magnetic pulses, electric current, or light to modify the function of the nervous tissue [1,2,3,4,5,6,7,8,9,10]. Chemical stimulation, although targeting specific receptors, lacks a millisecond scale of precision that defines the normal activity of the nervous tissue [2]. Transcranial magnetic stimulation is a repetitive neuromodulatory technique that applies magnetic pulses noninvasively and is valuable therapy in resistant cases of major depression, anxiety, and phobias [3,4]. Optogenetics is the application of light to selectively target genetically engineered neurons that respond to various wavelengths of light [5]. Although a valuable and promising research tool that portends a new therapeutic era, optogenetics is currently not applied in clinical practice. Electrical stimulation is the primary technique of invasive neuromodulatory applications used in various human treatment protocols. Means of transferring electric current to the nervous tissue differ according to the stimulation site: deep brain stimulation (DBS), spinal cord stimulation, vagal nerve stimulation, peripheral nerve stimulation, and cortical stimulation [6,7,8,9,10].

Deep brain stimulation (DBS) was established as a modern, non-destructive neuromodulatory therapy for the treatment of patients suffering from Parkinson’s disease in the early 1990s [11]. DBS is a technique that stimulates deep-seated nuclei in the telencephalon, diencephalon, and mesencephalon. Since its introduction, this technique has become one of the most effective treatment options for patients with advanced Parkinson’s disease, essential tremor, or generalized dystonia [6]. With the current state-of-the-art DBS techniques, more selective and directed stimulation through the multiangled arrangement of contacts is possible [12]. Although a very effective therapy, the exact mechanism of action of DBS is still unsettled, and few suggested mechanisms are constantly debated in the literature, see the review by Chiken [13].

As a relatively new therapy, DBS clinical application boundaries are continually expanding [14,15,16]. Experimental DBS of various animal models is used to search for new brain targets for diseases already successfully treated by electrical stimulation [17]. In the same manner, animal DBS models are employed to explore the possibility of applying DBS in the treatment of diseases so far not established as amenable to treatment by electrical stimulation [18,19]. The most promising candidate diseases for treatment by DBS are primary arterial hypertension (PAH) and obesity, which have pathogeneses stemming from failed neural control [20,21,22,23].

Long-term PAH is a major risk factor for coronary heart disease, heart failure, or stroke, responsible for 13% of deaths globally [24]. In 2015 high blood pressure affected approximately 1.13 billion people or 22% of the global adult population [25,26]. Because the disease burden of PAH is becoming more severe at an accelerating pace, expanding treatment options would be of immense value.

Contemporary PAH management options include lifestyle changes, pharmacological treatment, and non-pharmacological interventions. Non-pharmacological interventions mainly reserved for drug-resistant hypertension are carotid baroreceptor stimulation and percutaneous renal artery denervation [27,28]. Baroreceptor stimulation is applied to baroreceptors in the carotid sinus to inhibit the baroreflex and decrease the sympathetic tone reaching the heart and vasculature [27]. The percutaneous renal artery denervation procedure selectively ablates small sympathetic nerves around renal arteries to reduce salt and water retention [28].

Numerous scientific studies indirectly support the hypothetical potential of DBS to correct high blood pressure [27,28,29,30,31,32,33,34,35]. To provide a few examples, up to 44% of PAH could be due to increased sympathetic nervous system tone, as is evidenced by studies assessing the concentration of catecholamines in plasma or norepinephrine spillover in urine [29]. Solid indications suggest that abnormal sympathetic renal innervation could be partly responsible for the pathogenesis of PAH [34]. The effectiveness of baroreflex stimulation and renal denervation procedures, both affecting nervous regulation of blood pressure circuitry, shows that the neurogenic component in the pathogenesis of hypertension could be a major factor determining disease severity and drug resistance [27,28,35]. Therefore, applying DBS in the treatment of patients suffering from drug-resistant hypertension could be beneficial.

In non-survival experiments, applying electric current to the caudal ventrolateral medulla (CVLM) leads to profound hypotensive effects [36]. CVLM is a major relay center of the baroreflex arc that directly inhibits the rostral ventrolateral medulla, the main nucleus driving the sympathetic tone [37]. To our knowledge, long-term CVLM electrical stimulation in awake and freely moving rats hasn‘t been reported in the literature. Studies of brain stem stimulation have introduced the notion that long-term stimulation of the lower medullary region could lead to grave neurological compromise or even death [38,39]. Based on the facts mentioned above, our study aimed to develop and test in vivo, a remotely programmable brain stimulator combined with an invasive blood pressure monitoring system dedicated to DBS in the lower medullary region. In this paper, an emphasis on the survivability of animals, monitoring of neurological compromise, and patency rates of catheters was attempted.

## 2. Materials and Methods

### 2.1. Design of the Device

**Figure 1:**
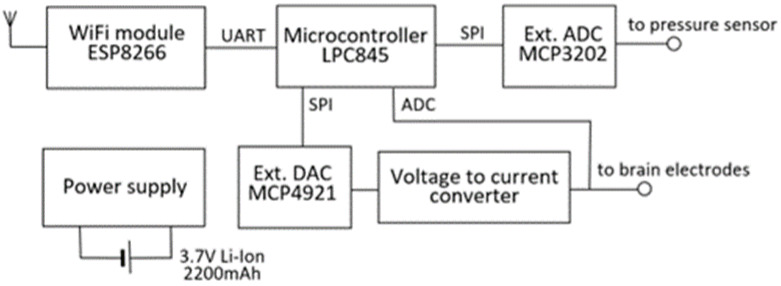
Parts of the measurement system.

The device consists of a wireless data transmission module and a measurement element (Figure 1). Wireless data transmission is based on the low-cost commercially available microchip (ESP8266 chip, Espressif Systems, Shanghai, China). A complete ESP8266 module with an integrated antenna, radio frequency (RF) circuit, and flash memory was used. Blood pressure measurement and brain stimulation were controlled by a microcontroller (LPC845, NXP Semiconductors). The microcontroller’s internal analog-to-digital converter (ADC) was used to record brain stimulation pulses in vivo. In contrast, the device’s external ADC and digital-to-analog converter (DAC) chips were used to measure blood pressure and generate brain stimulation pulses, respectively. The universal asynchronous receiver–transmitter (UART) interface was used for data and command exchange between the ESP8266 and LPC845. The power supply circuit generates 5 V and 3.3 V from a 3.7 V li-ion battery with a capacity of 2200 mAh. The blood pressure measurement circuit consists of a pressure sensor (BPS130, Bourns, Inc., Riverside, CA, USA) and external ADC (MCP3202, Microchip Technology Inc., Chandler, AZ, USA). A serial peripheral interface (SPI) was used for data exchange between LPC845 and the pressure measurement circuit. According to the sensor’s datasheet, the sensor’s zero drift and sensitivity drift should be minimal, and no recalibration process should be necessary for the usual physiological and pathological ranges of blood pressure [40].

The brain stimulation circuit is capable of generating an electric pulse of any arbitrary shape. The circuit consists of a voltage pulse generator and a voltage-to-current converter. Voltage pulse generation was performed by an LPC845 microcontroller and a DAC chip (MCP4921, Microchip Technology Inc., Chandler, AZ, USA). The voltage-to-current conversion was achieved by connecting brain tissue resistance to the negative feedback link of the operational amplifier (AD8542, Analog Devices Inc., Wilmington, MA, USA). This study used a charged-balanced, non-symmetrical pulse shape. The operating range and resolution of stimulation parameters and other important features of the device are summarized in Table 1.

### 2.2. Data Recording and Analysis

**Figure 2:**
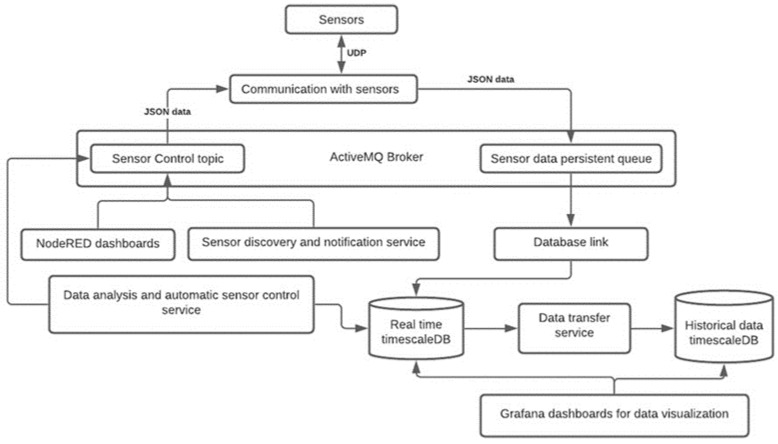
Software architecture.

**Figure 3:**
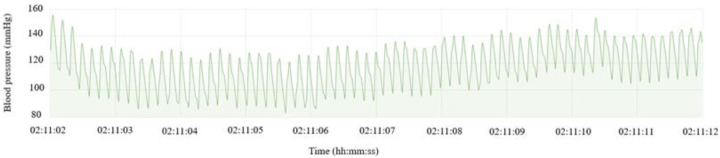
Ten seconds of calibrated data.

The data was recorded and analyzed with a PC running a Linux-based operating system with 8 Gb of RAM and a processing power of 2.4 GHz. A custom software solution was built for data collection and visualization (see Figure 2). The programming language Python was used for communication with the sensor. This component was designed so that it could handle large amounts of data. ActiveMQ was used as a broker for message exchange. NodeRED was used to create dashboards for manual sensor control (recording period, stimulation wave, etc.). A sensor discovery and notification service are responsible for sending broadcast messages to all sensors and monitoring the health of the sensors. Grafana dashboards were used to visualize real-time and historical data, see Figure 3. Helper services such as data transfer and daily data transfer were written with Python. Since the amount of data is large enough to put pressure on the database (100 data points per second from one sensor), a historical database was used to reduce the load on real-time data. For in-depth analysis and simulations, historical data were used. A daily data transfer job was created for transferring data from real-time and historical databases. A helper service was also responsible for removing data from the real-time database.

The data analysis component is responsible for pulse measurement, breathing rate measurement, and automatic sensor control. The pulse and breathing rates were measured by smoothing data using Gaussian kernels with different kernel bandwidths (20 for breathing rate extraction and 10 for pulse rate smoothing). The changes in blood pressure could also be monitored and stimulation parameters could be adjusted automatically in a closed-loop manner.

### 2.3. Device Peripherals

**Figure 4:**
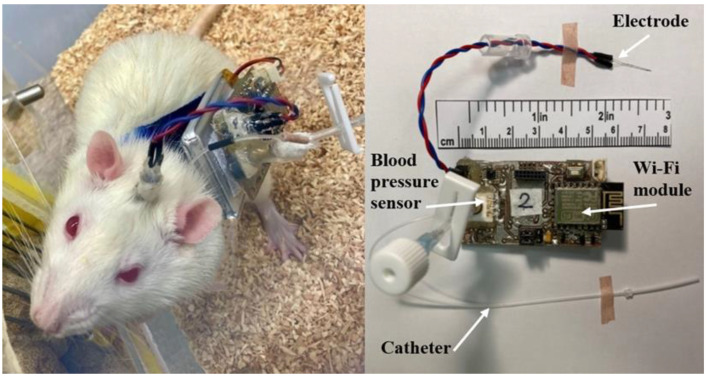
A Wistar rat equipped with the device is shown on the left. The device with an electrode, Wi-Fi module, blood pressure sensor, and catheter system is shown on the right.

The arterial catheters (Figure 4) were made of a polyethylene tube (Smiths Medical International Ltd., external diameter 0.96 mm, internal diameter 0.58 mm) with additional ports to replace lock solutions and sensor calibration. An anchor was fixed at 24–26 mm from the catheter tip and acted as a stopper and a securing device that positioned the catheter tip at the junction of the common carotid artery and aortic arch. Either 500 IU heparin/99.7% glycerol or 500 IU heparin/50% dextrose solutions were used for catheter lock solutions.

Electrodes made of stainless-steel wire of 100 µm in diameter with polyamide insulation of 5 µm (Goodfellow Cambridge Ltd., Huntingdon, UK) were used. The electrodes were produced in a bipolar fashion with 200 µm overhang between the tips.

The jackets for the rats were made from neoprene fabric with Velcro straps, and the device case was 3D printed (Ender-3 Pro, Creality) from polylactic acid plastic (Fiberology).

### 2.4. Procedure

All study protocols and experiments followed current European Union animal research regulations. Experiments were approved by Lithuanian institutional authorities (State Food and Veterinary Service No. G2-128).

The device’s performance was tested while experimenting on ten male Wistar rats aged 12–16 weeks, weighing 340–425 g, obtained from our in-house breeding colony (Vilnius University, Life Sciences Center, Lithuania). The rats were housed individually under controlled conditions with 12 h light/dark cycles, and drinks and food allowed ad libitum. Seven days before the implantation procedure, the animals were equipped with a jacket and a case simulating the device size to enable adaptation to restrained stress.

Surgical interventions were performed under sevoflurane (Baxter) anesthesia (3–4% in a mixture of 100% O_2_ administered through a nose cone). Catheter and electrode implantation procedures were performed in one session under a surgical microscope with ×6.4 magnification. Magnification allowed smaller surgical wound openings, better visualization and preservation of the vagal nerve and omohyoid muscle, as well as better control of electrode entrance. After the rats were anesthetized, their pain reflexes checked, and three applications of povidone-iodine solution (Egis Pharmaceuticals) given, two 1-cm-long median incisions in the vertex and suprasternal (anterior neck) regions were made. The device’s catheter filled with lock solution was inserted and tunneled subcutaneously between incisions through the left side of the neck. Caution was taken not to damage the left external jugular vein during the tunneling of the catheter. Approximately 0.5 cm of the left common carotid artery was bluntly dissected and exposed with two sutures proximally and distally. During dissection, the omohyoid muscle was left in a lateral position. Special care was taken while exposing the artery not to damage small vagal nerve branches that usually run over the carotid artery. The distal end of the artery was tied off, and a 2 mm longitudinal incision in the ventral wall of the carotid artery was made while tension was kept on the loose proximal suture. This technique allows almost bloodless catheter implantation and is the most challenging part of surgery to master. The catheter was inserted until the anchor reached the incision in the carotid artery, then the catheter was tied to the artery with two additional sutures. If the catheter’s intravascular part is too long, the catheter tip will occlude the aorta, and the animal will die. If the catheter is too short, it will become blocked by a clot and no pressure data will be gained. During the study, the observation was made that the length of the catheter’s intravascular part depended on the animal’s distance from the base of the tail to its nose. This could be a more accurate approximation than weighing the animal, as is usual practice in other laboratories. After implantation, the neck wound was closed with 5-0 sutures.

**Figure 5:**
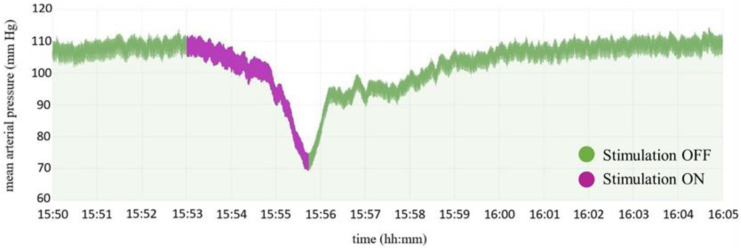
Mean arterial pressure change during the stimulation period intraoperatively marked in purple.

After the placement of the catheter, animals were put in a stereotaxic apparatus (Narishige Scientific Instrument Lab). Arterial blood pressure was continuously recorded during the electrode placement procedure. The already made vertex incision was used for electrode implantation; thus, additional care should be taken while manipulating the electrode or high-speed drill not to damage the already inserted and tunneled catheter. Because survival surgery was attempted, an indirect transcerebellar route was chosen for the electrode implantation instead of direct open implantation where the cerebellum should be partially removed to expose the IV ventricle. The skull was positioned to be level between Bregma and Lambda. A trephination of approximately 3 mm in diameter was drilled on the occipital ridge region on the right side. Two additional holes were drilled in the skull for dental screws, anterior to the trephination and posterior to the lambdoid suture. The coordinates of CVLM were chosen in accordance with Paxinos and Goodchild and were the following: −4.40 mm anteroposteriorly, 2.10 mediolaterally, and 9.90 dorsoventrally from Lambda [41,42]. After advancing the electrode tip to 8.5 mm in the z-axis, a stimulation protocol was implemented. Blood pressure response to electrical stimulation was recorded at multiple sites while lowering the electrode in steps of 0.1 mm. The implantation site was verified by two consecutive stimulation trains of 3 s (100 μA, 50 Hz, 0.1 ms) that caused the arterial pressure to drop at least 20 mm Hg (Figure 5). At least 1 min was given before applying the stimulus at the next location. No attempt was made to lower the electrode below 10.5 mm in the z-axis. After the accurate placement of the electrode, a head post made of dental micro-hybrid composite (FlowX, ORBIS) was affixed.

### 2.5. Postoperative Period

The postoperative and data collection period lasted continuously 24 h per day for two weeks. Close monitoring for abnormal postures and behaviors and administration of analgesia took place for 24 h after the procedure. Catheter patency was maintained by replacing lock solutions once per day with the known filling volume of the system. Blood pressure recordings were made with lock solutions in place, and no substitutions of heparinized saline were made. For lock solutions, either a heparinized 50% dextrose solution or a heparinized 99.5% glycerol solution was used. Five hundred IU of heparin per 1 mL of solution was added. Animals were closely monitored for neurological compromise by a staff veterinarian. Catheter patency status was determined as non-patent when there was evidence of loss of discernible blood pressure waves on the monitor. On the 8th day of the experiment, a chronic stimulation (50 μA, 50 Hz, 0.1 ms) was initiated that continued for the next seven days. The device pressure output (in mV) was calibrated to known-value pressure points of 0, 50, 100, 150, and 200 mm Hg using a custom pressure gauge kit. Calibrations were done immediately before implantation on the first day of the experiment, in the middle of the experiment on the 7th day, and at the end on the 14th day.

### 2.6. Data Analysis

Data analysis was done with R and MS Excel. To assess the stability of the device measuring circuitry, the one-factor analysis of variance (ANOVA) was used to analyze the difference in calibrated blood pressure output (in mV) on the 1st, 7th, and 14th days of the experiment. Mean blood pressure was averaged by extracting data from the SQL database (PostgreSQL). The blood pressure data gained from the first four days were not used for the analysis as rats were recovering from surgery and blood pressure curves were unstable. Mean blood pressure was averaged on days 5–7 (“stimulation OFF” period) and days 8–14 (“stimulation ON” period). A dependent Student *t*-test was used to analyze the difference in mean blood pressure between the “Stimulation OFF” and “Stimulation ON” periods. Only data gained from rats that survived the whole experimentation period without neurological or behavioral compromise were used in the data analysis. Statistical significance was taken at *p* < 0.05. Data were expressed as the mean ± standard deviation of the mean.

## 3. Results

### 3.1. Survivability and Neurological Compromise

Nine of the ten rats (90%) used in the study survived the surgery and regained wakefulness. One rat (10%) died during the procedure when the electrode had been advanced only 6 mm in the dorsoventral coordinate. The autopsy revealed an unexpectedly high-riding aortic arch and the catheter occluding the aorta. One rat regained wakefulness without evident neurological compromise and was hemodynamically stable, only to die eight hours later. The autopsy revealed a minor, 3 mm hemorrhagic lesion in the dorsal medulla in the path of the electrode. One rat had transient cerebellar symptoms: ataxic movements of the right side with no evident weakness that subsided in 48 h. The other seven rats (70%) were free of any apparent neurological compromise during the entire postoperative period.

### 3.2. Blood Pressure Recording

The mean blood pressure of rats during the “Stimulation OFF” period was higher (93.12 ± 5.33 mm Hg) than the mean blood pressure of rats during the “Stimulation ON” period (86.64 ± 6.47 mm Hg). A *t*-test for dependent samples showed that this difference was statistically significant, dF = 7, *p* = 0.004.

**Figure 6:**
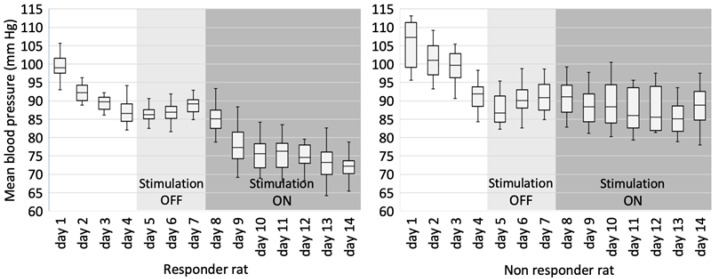
Typical graphs show a change in mean arterial blood pressure during two weeks of the experiment: response to stimulation on the left side and lack of response on the right side. “Stimulation OFF” and Stimulation ON” periods are shown in different shades of grey.

During the experiment, a clear distinction could be made between rats that had and had not responded to stimulation. A typical blood pressure pattern in animals that responded to stimulation is presented on the left side of Figure 6. Note the decrease in mean blood pressure when chronic stimulation on the 8th day was turned on. Note also, an acute rise in the mean blood pressure during the first three days after surgery, as this is related to the recovery and healing process postoperatively. Five out of eight rats (62.5%) that survived two weeks showed a similar blood pressure pattern, while the three rats (37.5%) did not respond to stimulation and had a blood pressure pattern similar to the one demonstrated on the right side of Figure 6.

### 3.3. Zero and Sensitivity Drift

**Figure 7:**
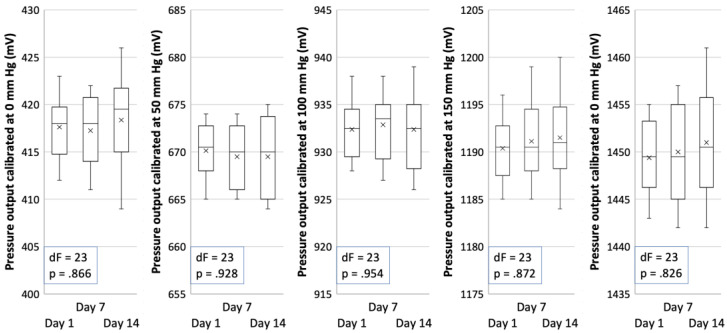
Device calibration data showing blood pressure output in mV on different experimental days. dF and *p* values of ANOVA are presented in the chart.

Device calibration data are illustrated in Figure 7. The ANOVA has shown that at all pressure points where the calibration of the device was performed, there is no significant difference between calibrated blood pressure output (in mV) and the day the calibration was done during the study, meaning that there is no significant device sensitivity drift.

### 3.4. Device Performance

**Figure 8:**
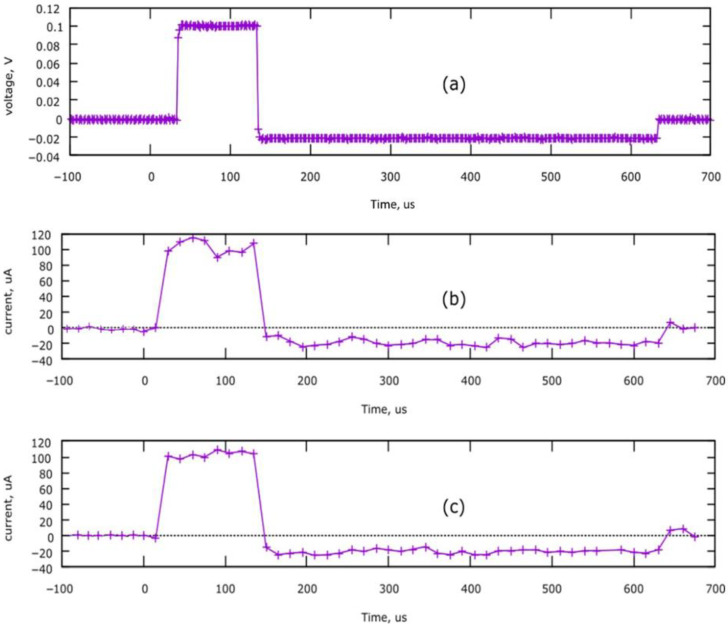
Brain stimulation pulse recordings. Pulse settings: Apulse = 100 µA, Tpulse = 100 µs. (**a**) Oscilloscope recording on 1000-ohm resistor; (**b**) Internal microcontroller’s ADC recording on 1000-ohm resistor; (**c**) In vivo internal microcontroller ADC recording.

Recordings of brain stimulation pulses are illustrated in Figure 8. Plot (a) is a voltage recorded with an oscilloscope on a 1000-ohm resistor; the current can be calculated by dividing the voltage by the resistor value. Plots (b) and (c) were recorded by the ADC of the internal microcontroller, and current value was calculated as a voltage drop on the R7 resistor. Plot (c) was recorded during in vivo brain stimulation. Note the similarities of simulation pulses between three different methods of recording.

### 3.5. Catheter Patency Rates

The mean duration of catheter patency filled with heparinized dextrose solution was 125.05 ± 22.54 min. The patency of the catheters filled with heparinized glycerol solution was stable for 24 h until daily catheter maintenance, and replacement of the lock solution was performed according to the study protocol. Non-patency of the glycerol-filled catheters was very rare. In all instances, it happened during the first day after the surgery when blood pressure perturbations were evident. As catheters filled with lock solutions outlasted the catheters filled with dextrose solutions by quite a margin, the application of statistical tests was not reasonable.

## 4. Discussion

Although this is a preliminary/pilot study that seeks to test the device performance in vivo, the results of the CVLM stimulation are encouraging, as five rats responded to stimulation with a discernible hypotensive effect. This effect was less notable than in studies of acute, non-survival surgery designs [36,43]. It is known that anesthesia facilitates depressor pathways in the brain [44,45]. Although O’Callaghan et al. showed considerable hypotensive effect while stimulating periaqueductal gray matter (PAG) in anesthetized rats, they failed to show the same results when the rats were awake [45].

In the literature, we have not found any successful chronic CVLM stimulation studies that show the survivability of the animals after the implantation and, at the same time, demonstrate the effectiveness of stimulation. Studies involving stimulation or recording with lower medullary electrodes have usually been performed in a surgical manner that does not assure survival [46,47,48]. A few publications were found presenting research concerning the stimulation of the brain stem with electrodes in survival surgery, but none with the position of the electrodes in such a posterior location [49,50,51]. This could be due to a notion that lower medullary lead implantation could be dangerous and prone to severe neurological complications in the region of the ‘no man’s land of neurosurgery’ [38,39]. This is the first study to show considerable survivability of animals with stimulating electrodes implanted in the CVLM.

CVLM DBS would be difficult to translate to human trials. The lowest point of the human brainstem axis where electrodes were inserted was the locus coeruleus region and parabrachial complex, both in the lower pons [52,53]. Those successful implantations were done in the pre-DBS era by courageous neurologists and neurosurgeons and are not possible nowadays due to a lack of reproducible findings and a drift to more superior targets that could be easier to reach without risking severe complications [54]. Second, the technology of DBS may not have advanced enough to allow such a delicate structure as the CVLM to be targeted.

Clinical data exploring the DBS treatment effect on drug-resistant hypertension comes from a few studies targeting PAG [20,55,56,57]. From a physiological theoretical perspective, CVLM, although a difficult-to-reach target, has two inherent advantages over PAG. First, CVLM has no known direct neuroendocrine, nociceptive, or integrative behavioral function. Its primary function is to relay information from NTS to the RVLM in a reflex manner [45]. Second, being the lowest inhibitory center of the autonomic nervous system, CVLM should influence different pathogenetic circuitry of arterial hypertension. No matter the cause of hypertension, be it obesity-induced, salt-sensitive, or hypertension caused by sensitization of autonomic pathways, CVLM stimulation should be able to lower arterial blood pressure effectively [58].

An analysis of blood pressure calibration data did not show significant sensitivity drift at any calibration point during the experiment. This is in accordance with the datasheet for the BPS130 sensors [40]. The device is stable and can be used for further research.

Direct blood pressure in laboratory animals can be recorded by an implantable radio telemetry transducer or an external transducer attached through a swivel to a catheter system [59]. Laboratories use many custom-made pulse generator systems worldwide [60,61,62]. This device combines both functions: wireless blood pressure telemetry and an electric pulse generator with the possibility of closed-loop stimulation. To our knowledge, this is the first device that combines blood pressure telemetry and a brain stimulator in one device that is sufficiently small to mount on the animal. Although appearing relatively robust, no animal demonstrated restrained behaviors (changes in postures, eating, or drinking habits) during the study period (Figure 4).

It is a common practice that studies presenting custom stimulators fail to acknowledge the testing part of the created device or the testing being only rudimentarily performed with 1000-ohm resistors [60,61]. This device was tested in vivo to check whether cerebral biopotentials as high as 100 µV could interfere with introducing an electric current into neural tissue. Testing did not show that the device worked differently in vivo compared to conditions in which a resistor was used. (Figure 8).

Catheter patency rates showed results that were comparable to those demonstrated by Luo et al. [63]. In this research paper, the patency of the catheter was determined visually by assessing whether flushing of the system was still possible. In this study, the patency of the catheters was determined by evaluating whether discernible blood pressure waves were evident. In this regard, catheters filled with heparinized glycerol solutions were patent for up to 24 h when daily replacement of lock solutions was done following the study protocol.

All blood pressure recordings were done while the catheter system was filled with lock solutions, acting not only to prevent clotting but as a transfer agent of pressure force to the sensor. In our study, blood pressure waves were comparable to high-fidelity blood pressure recordings in other studies [64]. Even without a normal pressure wave signal, determination of mean arterial blood pressure is possible as long as a discernible pressure wave is evident, indicating that catheter patency is maintained [59].

## 5. Conclusions

The device presented in this study is stable and provides reproducible measurements of blood pressure while at the same time stimulating targets in the medullary region. The equipment can be used as a novel solution for testing various targets that could be implicated in blood pressure control in animal models.

More studies with groups, including sham controls, are needed to demonstrate the effect of CVLM stimulation on blood pressure. Future studies should also include not otherwise healthy Wistar rats, but instead rats prone to hypertension, such as spontaneously hypertensive rats. Furthermore, the effect of bilateral CVLM stimulation should also be tested as this could cause a reinforced effect on pressure change. Future studies could even include other targets in the lower medullary region, such as the rostral ventrolateral medulla. As a cardioaccelerator center, this particular target could be used to treat patients prone to vasovagal syncope. Further, new opportunities could be explored for treating conditions such as a vegetative state after severe traumatic brain injury or other brain tissue insult.

## Figures and Tables

**Table 1:** Summary of the device parameters and features.

Pulse amplitude	0–140 µA, steps of 1 µA
Pulse duration	40–300 µs, steps of 1 µs
Pulse repetition period (frequency)	2–500 ms (2–500 Hz), steps of 1 ms
Weight (with jacket, case and battery)	55 g
Dimensions (with case)	5.5 × 3 × 2 cm
Operation time with one battery	20 h with continuous stimulation parameters: 100 μA, 50 Hz, 0.1 ms, and continuous data measurement and transmission
Programmable periodical sleep cycles for energy saving. In sleep mode, the WiFi module is not operational and blood pressure is not measured, while brain stimulation is always on.	
The pressure measurement period is software configurable and can be as little as 4 ms.	

## Data Availability

The data presented in this study are available on reasonable request from the corresponding author (Ž.C.).
